# Hydroquinine Enhances the Efficacy of Contact Lens Solutions for Inhibiting *Pseudomonas aeruginosa* Adhesion and Biofilm Formation

**DOI:** 10.3390/antibiotics13010056

**Published:** 2024-01-05

**Authors:** Sattaporn Weawsiangsang, Nontaporn Rattanachak, Sukunya Ross, Gareth M. Ross, Robert A. Baldock, Touchkanin Jongjitvimol, Jirapas Jongjitwimol

**Affiliations:** 1 Biomedical Sciences Program, Faculty of Allied Health Sciences, Naresuan University, Phitsanulok 65000, Thailand; sattapornw63@nu.ac.th; 2 Biology Program, Faculty of Science and Technology, Pibulsongkram Rajabhat University, Phitsanulok 65000, Thailand; nontaporn.r@psru.ac.th (N.R.); touchkanin@psru.ac.th (T.J.); 3 Biopolymer Group, Department of Chemistry, Faculty of Science, Naresuan University, Phitsanulok 65000, Thailand; sukunyaj@nu.ac.th (S.R.); gareth@nu.ac.th (G.M.R.); 4 Centre of Excellence in Biomaterials, Faculty of Science, Naresuan University, Phitsanulok 65000, Thailand; 5 School of Pharmacy and Biomedical Sciences, Faculty of Science and Health, University of Portsmouth, Portsmouth PO1 2DT, UK; robert.baldock@port.ac.uk; 6 Department of Medical Technology, Faculty of Allied Health Sciences, Naresuan University, Phitsanulok 65000, Thailand

**Keywords:** adhesion, biofilm, contact lens solution, hydroquinine, microbial keratitis, *Pseudomonas aeruginosa*

## Abstract

*P. aeruginosa* is one of the most common bacteria causing contact lens-related microbial keratitis (CLMK). Previous studies report that disinfecting solutions were ineffective in preventing biofilm formation. Solutions containing novel natural agents may be an excellent alternative for reducing the risk of CLMK. Here, we investigate the disinfecting properties of hydroquinine in combination with multipurpose solutions (MPSs) to prevent *P. aeruginosa* adhesion and biofilm formation. We examined the antibacterial, anti-adhesion, and anti-biofilm properties of hydroquinine-formulated MPSs compared to MPSs alone. Using RT-qPCR, hydroquinine directly affected the expression levels of adhesion-related genes, namely, *cgrC*, *cheY*, *cheZ*, *fimU*, and *pilV*, resulting in reduced adhesion and anti-biofilm formation. Using ISO 14729 stand-alone testing, hydroquinine met the criteria (>99.9% killing at disinfection time) against both *P. aeruginosa* reference and clinical strains. Using the crystal violet retention assay and FE-SEM, MPSs combined with hydroquinine were effective in inhibiting *P. aeruginosa* adhesion and destroying preexisting biofilms. This report is the first to highlight the potential utility of hydroquinine-containing formulations as a disinfecting solution for contact lenses, specifically for inhibiting adhesion and destroying biofilm. These findings may aid in the development of novel disinfectants aimed at combating *P. aeruginosa*, thereby potentially reducing the incidence of CLMK.

## 1. Introduction

In recent decades, there has been a global increase in the number of individuals wearing contact lenses [1,2]. These lenses are preferred by those who desire clear vision without the need for spectacles [2]. While generally well-tolerated, contact lens wearers may experience a range of complications [2,3], particularly corneal ulcers associated with overnight wear of contact lenses [4]. In addition, contact lens use is a significant risk factor for developing corneal infections (microbial keratitis), with incidence rates varying by wear modality. These rates range approximately from 2 to 20 cases per 10,000 wearers annually [5]. In severe instances, microbial keratitis can lead to permanent vision loss [5]. *Pseudomonas aeruginosa* is the most common pathogen that causes contact lens-related microbial keratitis (CLMK) [6,7,8,9], accounting for about 55–59% of the CLMK-related isolates [8,9]. Furthermore, *P. aeruginosa* infection is associated with higher ocular severity, e.g., corneal perforation within 72 h [10]. According to the World Health Organization (WHO), corneal blindness resulting from microbial keratitis is emerging as a prominent cause of visual disability [11,12]. Moreover, multidrug-resistant (MDR) *P. aeruginosa* has been classified as one of the most concerning pathogens by the WHO [13]. Importantly, biofilm formation is the main virulence factor that is associated with increased severity of microbial keratitis [14]. For example, *P. aeruginosa* biofilm formation promotes resistance to antibiotic treatments—biofilm formation is stimulated by the quorum-sensing (QS) system [15]. Interestingly, biofilm-mediated antibacterial resistance requires the flagella, pili, and other adhesins to trigger the biofilm formation [16]. It is evident that bacterial biofilms facilitate prolonged contamination of contact lenses and the persistence of microbial contamination in contact lens cases [17]. According to numerous epidemiological studies, it has been demonstrated that the annualized incidence of CLMK significantly increases with overnight and/or extended lens wear compared to daily wear (from a 5- to 10-fold increase) [18]. In addition, other factors can also contribute to risk, such as patient compliance and hand hygiene, the type of lens care solution used, and the microbial contamination of the lenses or lens cases [18]. Although contact lens solutions meet the international ISO 14729 and FDA criteria for adequate antimicrobial efficacy, they are only subjected to assessment against selected microbial reference strains [19,20,21,22]. Moreover, the antimicrobial activity does not guarantee efficacy against clinical strains [22]. In addition, commercially available disinfecting solutions may be ineffective against biofilms [20,21,22]. Therefore, the prevention or elimination of biofilm formation on contact lenses is now necessary for developing new strategies to reduce the risk of biofilm-associated ocular infection [23]. New agents derived from natural products that prevent biofilm formation may be an excellent option to limit or reduce the risk of contact lens contamination.

Many natural compounds have been used for ocular therapies because of their anti-infective and anti-inflammatory properties [24]. Interestingly, hydroquinine is a natural substance that has been shown to possess antimicrobial properties [25,26]. Kraikongjit et al. (2018) [25] and Jongjitvimol et al. (2020) [26] have previously demonstrated that hydroquinine is one of the key substances in the ethanolic nest entrance extracts from *Tetrigona apicalis* that exhibit antibacterial, antifungal, and anti-proliferative activities [25,26]. Moreover, hydroquinine has anti-malarial and anti-melanin activities, which may be used for reducing light-brown skin patches and skin discoloration associated with pregnancy [27]. In clinical settings, hydroquinine has been used to relieve nocturnal cramps in the Netherlands [28]. Recently, it has been shown that hydroquinine inhibits and kills both Gram-positive and Gram-negative bacteria, including *Staphylococcus aureus*, *Enterobacter cloacae*, *Escherichia coli*, *Klebsiella pneumoniae*, and, in particular, *P. aeruginosa* [29]. Furthermore, Jongjitwimol and Baldock proposed that hydroquinine has potential as an antimicrobial agent to target the MDR strains of *P. aeruginosa* [30]. In support of this, recent research has shown that hydroquinine showed anti-*P. aeruginosa* efficacy against both clinical drug-sensitive (DS) and multidrug-resistant (MDR) *P. aeruginosa* strains [31].

Rattanachak et al. (2022) used transcriptomics approaches and identified that the levels of several key transcripts were altered in *P. aeruginosa* ATCC 27853 when treated with 1.25 mg/mL hydroquinine for an hour [32], because this concentration has some molecular and functional effects against *P. aeruginosa* without a killing effect [29]. Interestingly, there were several differentially expressed genes that were significantly downregulated, relating to adhesion and biofilm formation processes, namely, *cgrC*, *cheY*, *cheZ*, *fimU*, and *pilV* (Table S1). We, therefore, hypothesized whether a half-MIC hydroquinine treatment could reduce the expression of the adhesion-related genes in *P. aeruginosa*. We also further investigated the antimicrobial efficacy of hydroquinine in combination with multipurpose solutions (MPSs) against *P. aeruginosa* strains. This investigation encompassed various aspects, including anti-bacterial activity, anti-adhesion efficacy, and anti-biofilm mass formation on contact lenses.

## 2. Results

### 2.1. Hydroquinine Inhibits P. aeruginosa Growth through Decreased Expression Levels of Adhesion-Related Genes

**Figure 1:**
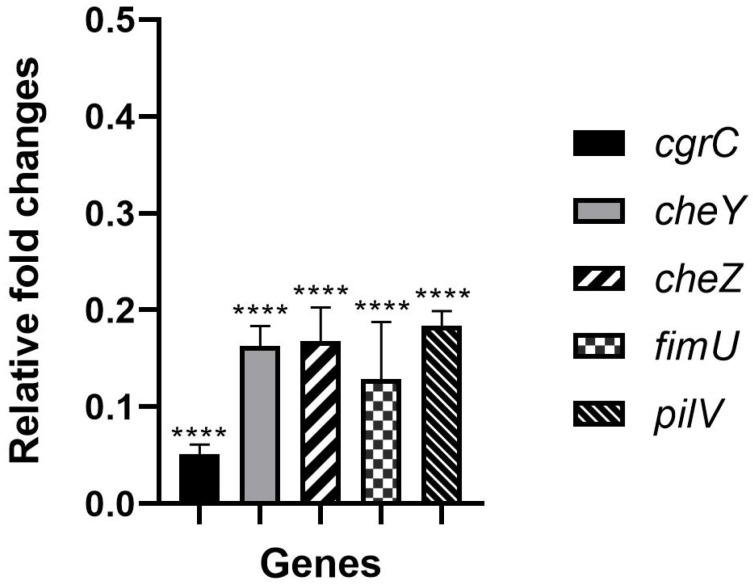
The relative expression levels of the adhesion-related genes treated with 1.25 mg/mL of hydroquinine for one hour in *P. aeruginosa* ATCC 27853 compared to the corresponding untreated control. Asterisks **** denote *p* < 0.0001. The triplicate data are presented as mean ± SD.

To validate the expression of the adhesion-related *genes* in response to hydroquinine, we identified that at 1.25 mg/mL, hydroquinine reduces adhesion-related gene expression in *P. aeruginosa* ATCC 27853 (Figure 1). The quantitative reverse transcription polymerase chain reaction (RT-qPCR) result shows significant reductions in the mRNA expressions of *cgrC*, *cheY*, *cheZ*, *fimU*, and *pilV* genes of 0.05 ± 0.02, 0.16 ± 0.04, 0.17 ± 0.06, 0.13 ± 0.10, and 0.18 ± 0.03 -fold, respectively, compared to the corresponding untreated control.

### 2.2. MPSs Inhibit P. aeruginosa Growth Using the ISO 14729 Criteria

The two commercial MPSs used in this study were Opti-free^®^ Replenish^®^ solution containing 0.001% POLYQUAD^®^ and 0.0005% ALDOX^®^ (MPS A) and Q-eye multipurpose solution containing 0.0001% polyhexamethylene biguanide (PHMB) (MPS B). The phosphate buffer saline (PBS) was used as an untreated control.

To investigate the disinfection efficacy of commercial MPSs, the log reduction in the bacterial growth was calculated. All commercial solutions studied met the ISO 14729 primary stand-alone criteria (3 log of reduction) [19] for bacterial efficacy at both 6 and 24 h contact time against *P. aeruginosa* ATCC 27853 and clinical *P. aeruginosa* strains.

**Figure 2:**
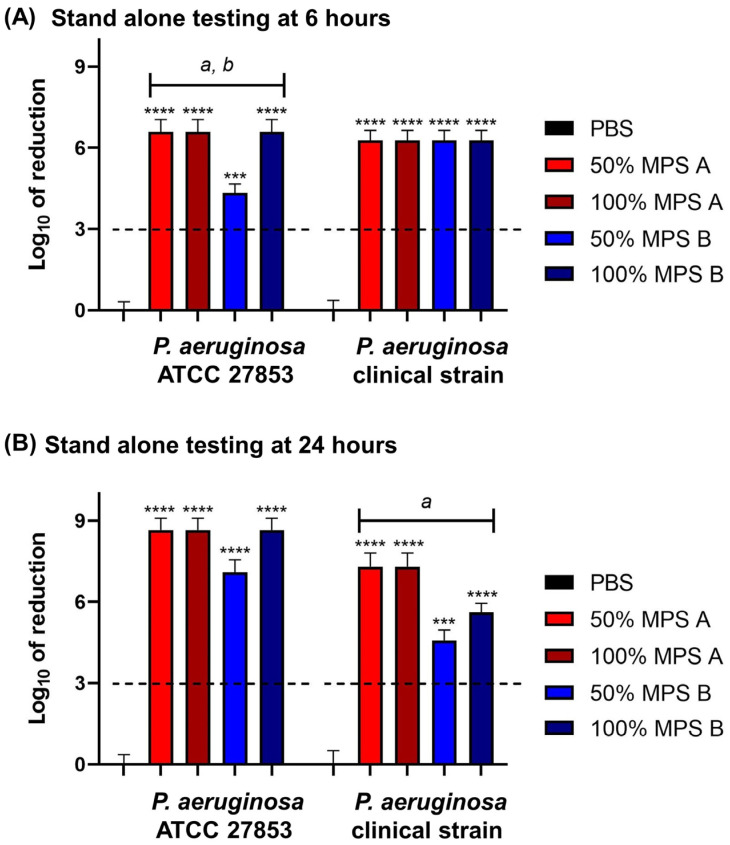
The log of reduction in the *P. aeruginosa* growth as a parameter of antibacterial efficacy of multipurpose solutions at (**A**) 6 h and (**B**) 24 h contact times compared to the corresponding untreated controls (PBS). The data are presented as mean ± SD. The dashed line represents the ISO 14729 criteria (3 log of reduction). The asterisk *** and **** symbols are *p* < 0.001, and *p* < 0.0001, respectively, compared to PBS at the same time point and within the same strain. Statistical differences among tested solution families: *a* is *p* < 0.05 for 50% MPS A vs. 50% MPS B, *b* is *p* < 0.05 for 50% MPS B vs. 100% MPS B at the same time point and within the same strain.

When we compared the log-fold reduction in *P. aeruginosa* strains after 6 h of disinfection time, the MPSs efficacies decreased with *P. aeruginosa* ATCC 27853. Specifically, 50% of MPS B significantly decreased in disinfection efficacy when compared to the original concentration. Moreover, 50% of MPS B had less efficacy compared to 50% of MPS A. However, there is no significant difference between MPS A and B against the clinical *P. aeruginosa* strain (Figure 2A). On the other hand, the efficacy of MPS B was reduced against the clinical *P. aeruginosa* strain with a 24 h contact time. The MPS B efficacy dramatically reduces at both half concentration and the original concentrations compared with MPS A efficacy. Moreover, it was observed that at 50% of MPS B, there was a significant decrease in disinfection efficacy compared to its efficacy at the original concentration (Figure 2B).

### 2.3. MPSs at Half Its Original Concentration Also Reduce P. aeruginosa Adhesion

To determine the anti-adhesion capacity of the MPSs, the solutions were challenged with *P. aeruginosa* strains and then assessed using the crystal violet retention method. The percentage of the residual biofilm formation was quantified and calculated to determine the inhibition of the adhesion as anti-adhesion efficacy (Table 1). Both MPSs had statistically strong anti-adhesion efficacy compared with the control (*p* < 0.0001). The percentages of anti-adhesion efficacy were between 89.76 and 91.89% in MPS A, and 75.05 and 86.83% in MPS B. Interestingly, MPS A at both 50% and 100% concentrations had more anti-adhesion efficacy than MPS B (Table 1). The 50% concentration of MPS A was more efficacious than the 50% concentration of MPS B (*p* < 0.0001 in both strains). Consistent reduced the concentrations, the original concentration of MPS A showed more adhesion inhibition than MPS B (statistically significant at *p* < 0.0001 in *P. aeruginosa* ATCC 27853 and at *p* < 0.01 in clinical *P. aeruginosa* strain). Furthermore, and as expected, the disinfection efficacy of the 50% concentration of MPS B was significantly decreased when compared with 100% of MPS B (*p* < 0.0001). Therefore, MPS A was further investigated at both 50% and 100% of its original concentration, combined with the hydroquinine solution using the same approach.

### 2.4. Hydroquinine Shows Comparable Inhibitory Activity to MPSs, and When Used in Combination, Damages Cell Structure

Next, we sought to determine the disinfection efficacy of a combination of hydroquinine with MPS A against *P. aeruginosa*. Two concentrations of hydroquinine were tested (half-MIC and MIC, 1.25 and 2.50 mg/mL of hydroquinine, respectively) for 6 and 24 h. The disinfection efficacy was determined by the calculating the log-fold reduction in the bacterial growth.

**Figure 3:**
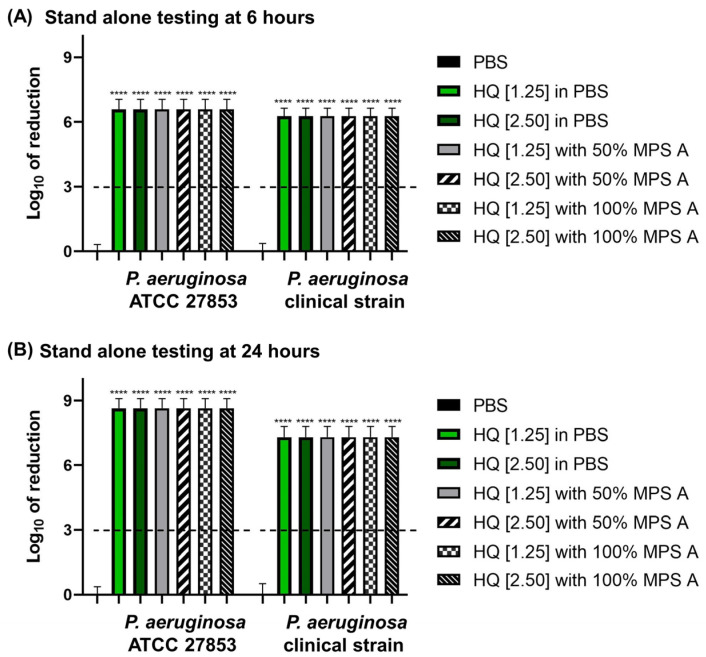
The log of reduction in the *P. aeruginosa* growth as a parameter of antibacterial efficacy of multipurpose solutions at (**A**) 6 h and (**B**) 24 h contact times compared to the corresponding untreated controls (PBS). The data are presented as mean ± SD. The dashed line represents the ISO 14729 criteria (3 log of reduction). The asterisks **** represent *p* < 0.0001 compared to PBS at the same time point and within the same strain.

Hydroquinine was as effective as commercially available MPSs. MPSs in combination with hydroquinine also demonstrated strong disinfection efficacy. All solutions tested met the ISO 14729 criteria. The results showed the log-fold of the reduction in the bacterial growth at more than a 6-log reduction (higher than 99.9999% killing) of both *P. aeruginosa* strains at 6 and 24 h contact times (Figure 3).

**Figure 4:**
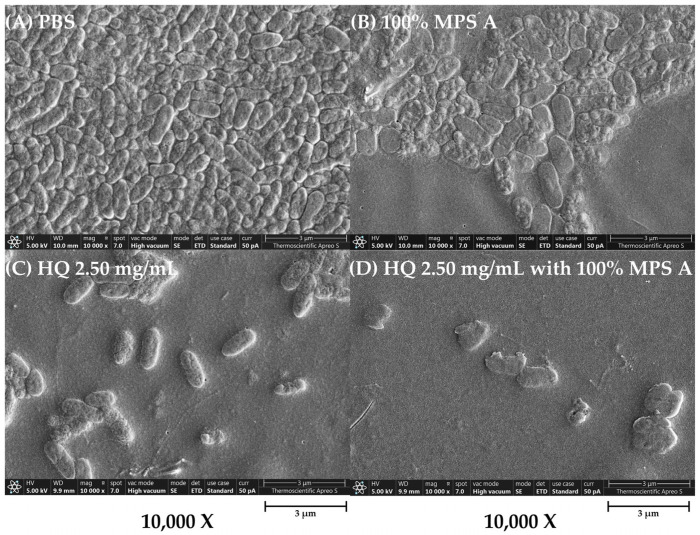
The structural characterization of *P. aeruginosa* ATCC 27853 as a representative strain in different tested solutions: (**A**) PBS, (**B**) 100% MPS A, (**C**) hydroquinine (HQ) 2.50 mg/mL, and (**D**) HQ 2.50 mg/mL with 100% MPS A. The images are presented at a magnification of 10,000× using the FE-SEM.

To further characterize the impact of hydroquinine on *P. aeruginosa* alone and in combination with MPSs, we examined changes in the bacterial structure of treated *P. aeruginosa* strains using a field emission scanning electron microscope (FE-SEM). The structure of *P. aeruginosa* treated with either a 100% concentration of MPS A, hydroquinine at 2.50 mg/mL, and hydroquinine at 2.50 mg/mL in combination with 100% MPS A is shown in Figure 4. The FE-SEM shows the untreated bacterial structure, including distinct borders between cells that are clear and uniform (Figure 4A). However, in *P. aeruginosa* treated with MPS A and/or hydroquinine solutions, the number of bacterial cells was reduced (Figure 4B–D). When a combination of hydroquinine and MPS A was administered, there were notable changes in the integrity of the cells, e.g., destroyed cell borders, changed morphological structures (irregular shapes), and unequal cell sizes (Figure 4D).

### 2.5. Hydroquinine with Commercial MPS Demonstrates the Synergistic Effect to Reduce P. aeriginosa Adhesion on Contact Lens Surface

**Figure 5:**
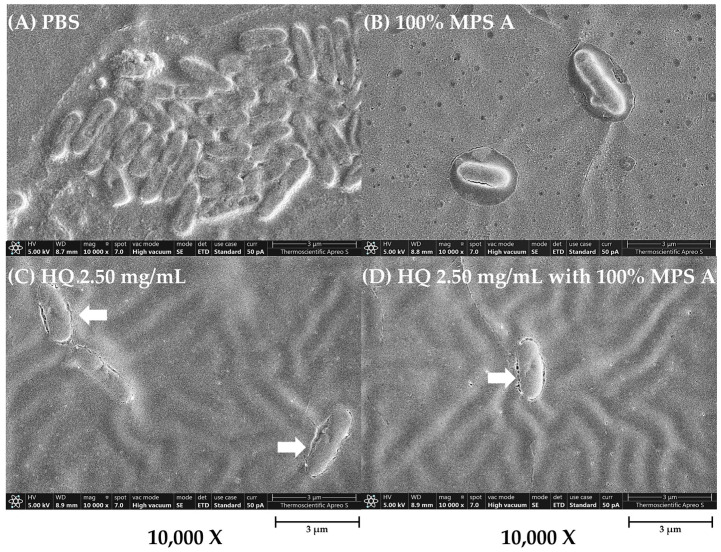
The adhesion of *P. aeruginosa* ATCC 27853 on contact lens surface as a representative strain in different tested solutions: (**A**) PBS, (**B**) 100% MPS A, (**C**) hydroquinine (HQ) 2.50 mg/mL, and (**D**) HQ 2.50 mg/mL with 100% MPS A. The images are presented at a magnification of 10,000× using the FE-SEM. The white arrow represents the damaged cell membrane.

We then sought to investigate the anti-adhesion properties of hydroquinine combined with MPS against the reference and clinical strains, *P. aeruginosa* ATCC 27853 and clinical *P. aeruginosa*, respectively. The percentage of anti-adhesion efficacy using the crystal violet retention assay is shown in Table 2. Additionally, we also tested whether hydroquinine alone and in combination showed disinfection efficacy on the surface of the contact lenses (Figure 5).

Hydroquinine at 1.25 and 2.50 mg/mL had anti-adhesion efficacy greater than 50% in both *P. aeruginosa* strains (between 54.84 and 59.56%). Interestingly, when using hydroquinine combined with MPS A, anti-adhesion was significantly enhanced (*p* < 0.0001) compared with hydroquinine alone. The percentage of the anti-adhesion efficacy of the combination was between 95.59 and 97.91% in *P. aeruginosa* ATCC 27853 and between 92.23 and 93.64% in the clinical *P. aeruginosa* strain (Table 2). Furthermore, the MPS A at half the original concentration combined with hydroquinine had higher anti-adhesion efficacy than the original MPS A manufacturer’s product, indicating the synergy between the MPS and hydroquinine (Table 1 and Table 2).

We then wanted to determine whether MPS formulations containing hydroquinine exhibit disinfection efficacy on contact lens surfaces. All tested solutions reduced *P. aeruginosa* growth and adhesion on contact lens surfaces at the disinfection time (6 h) using a standard testing (as described in Section 4.8). The cell structure of the *P. aeruginosa* ATCC 27853 strain was examined using FE-SEM (representative images are shown in Figure 5). In the PBS control, the microorganisms were tightly attached in packs on the contact lens surface with an ordered and dense cell-to-cell contact (Figure 5A). *P. aeruginosa* treated with 100% MPS A, hydroquinine alone, or the combination showed a reduced number of cells and bacterial adhesion (Figure 5B–D). Moreover, the cell membrane integrity also appeared to be altered (white arrow), showing an irregular shape.

### 2.6. MPS Formulations Containing Hydroquinine Reduce Biofilm Mass on Contact Lens Surfaces

To determine the disinfection effectiveness of MPSs containing hydroquinine, we also tested whether the combined solution destroys biofilm mass on contact lenses. We simulated the *P. aeruginosa* adhesion to allow the biofilm formation for 24 h and then evaluated the biofilm mass by using the FE-SEM. The biofilm mass observed in this study was a conglomeration of only *P. aeruginosa*, not other microorganisms.

**Figure 6:**
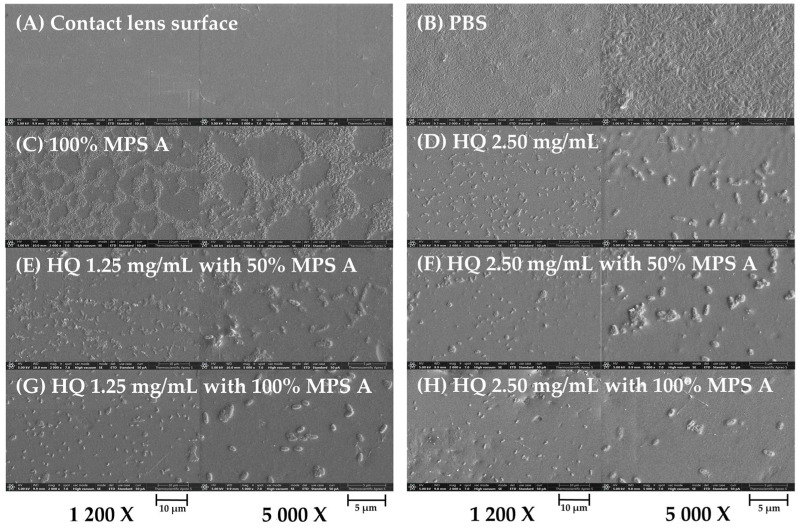
The biofilm mass of *P. aeruginosa* ATCC 27853 on contact lens surface as a representative strain in different tested solutions: (**A**) contact lens surface, (**B**) PBS, (**C**) 100% MPS A, (**D**) hydroquinine (HQ) 2.50 mg/mL, (**E**) HQ 1.25 mg/mL with 50% MPS A, (**F**) HQ 2.50 mg/mL with 50% MPS A, (**G**) HQ 1.25 mg/mL with 100% MPS A, and (**H**) HQ 2.50 mg/mL with 100% MPS A. The images are presented at magnification 1200× (left panels) and 5000× (right panels) using the FE-SEM.

When untreated, *P. aeruginosa* microbial communities are embedded in a 3D extracellular matrix (biofilm mass). The structure of the biofilm mass was compact and packaged together with tight cell-to-cell contacts (Figure 6B). MPS A, at the original concentration, destroyed a small quantity of the biofilm mass (Figure 6C). However, formulations containing hydroquinine demonstrated a profound disinfection efficiency in removing the biofilm mass from the contact lens surface. For example, the biofilm mass was broken down and dispersed when treated with either hydroquinine alone or in combination with MPS A (Figure 6D–H).

## 3. Discussion

Hydroquinine has been shown to be effective at killing both drug-sensitive and multidrug-resistant *P. aeruginosa* [29,31,32]. Hydroquinine attenuates *P. aeruginosa* growth by reducing flagella activity, pyocyanin production, and biofilm formation [32]. In this study, we hypothesized that hydroquinine might be efficacious at minimizing bacterial adherence to, and colonization of, contact lenses. As mentioned, *P. aeruginosa* is the most common pathogen that causes contact lens-related microbial keratitis (CLMK) [6,7,8]. The motility of *P. aeruginosa* is driven by two types of appendages, which comprise a single polar flagella and multiple type IV pili. The flagellum operates as a rotor and generates forward movement via hydrodynamic force [33,34,35]. In contrast, the type IV fimbriae or pili operate as linear actuators that pull the bacterium along a surface [33,34,35]. For *P. aeruginosa*, the type IV pili are pilin-containing filaments on the surfaces that are associated with adhesion, motility, microcolony formation and secretion of proteases, and colonization factors [35].

In this study, we present evidence that hydroquinine downregulates genes involved in *P. aeruginosa* adhesion ability. Using RT-qPCR, the mRNA expression levels of *cgrC*, *cheY*, *cheZ*, *fimU*, and *pilV* genes were significantly decreased in response to hydroquinine treatment (Figure 1). Interestingly, the expression of the *cgrC* gene was especially downregulated (relative expression levels of 0.05 ± 0.02 -fold). The *cgrC* gene encodes the *cupA* gene regulator C (CgrC), which controls the phase-variable expression of the *cupA* gene [36,37]. The *cup* gene cluster (chaperone-usher pathway), in particular, *cupA*, encodes the components of *P. aeruginosa* assembly factors of the fimbrial structure [38,39]. These factors facilitate surface attachment and motility and enable the formation of biofilm mass on abiotic surfaces [38,39]. Triggering *cgr* gene transcription results in the activation of *cupA* gene expression [37]. Therefore, the repression of *cgrC* gene by hydroquinine likely affects *cupA* gene expression, leading to the disruption of the fimbrial adhesins components in *P. aeruginosa*. Consistent with *cgrC*, the other genes, including *cheY*, *cheZ*, *fimU*, and *pilV*, were also significantly downregulated with hydroquinine treatment, which may also have impacted bacterial motilities and their adhesion process. For example, *cheY* and *cheZ* are related to chemotaxis [40]. The *cheY* gene encodes the two-component response regulator CheY, while the *cheZ* gene encodes the chemotaxis protein CheZ. Chemotaxis is the directed movement in response to changes in the chemical environment. Bacteria can respond to the chemical gradients using a chemosensory system coupled with flagella, fimbriae, or pili [41]. The CheY and CheZ proteins play a role in producing and transmitting the signals to flagellar motors, subsequently affecting bacterial motility [40]. For another example, the *fimU* and *pilV* genes encode type IV fimbrial biogenesis proteins, FimU and PilV, respectively [42,43]. FimU and PilV are proteins that play an important role in the biogenesis of type IV fimbrial proteins [42]. PilV possesses prepilin-like leader sequences [44]. FimU is required for both cleavage of the prepilin-like leader sequences and the subsequent methylation of the mature protein in the biogenesis and function of type IV fimbriae in *P. aeruginosa* [44,45,46]. This finding is the first identified evidence suggesting that hydroquinine inhibits the *P. aeruginosa* chemotaxis pathways by downregulating the expression levels of the *cheY* and *cheZ* genes. Furthermore, hydroquinine also reduces the biogenesis of bacterial surface organelles, i.e., type IV fimbria, likely affecting bacterial microcolony formation and colony expansion [35]. This is supported by previous research showing that deleting the appendage leads to deficiencies in cell attachment and growth [33]. Several studies reported that both flagella and type IV pili influence the initial stages of biofilm formation during the bacterial transition from a free-swimming planktonic state to a surface-associated state and, subsequently, microcolony formation [33,47,48]. Additionally, the bacterial appendages facilitate its binding to various surfaces and twitching motility on surfaces [38,42,49]. Therefore, we believe that hydroquinine might affect the motility of *P. aeruginosa* through these adhesion-related genes. This is consistent with the previous study showing that hydroquinine has strong anti-motility effects in *P. aeruginosa*, affecting both swimming and swarming abilities [32].

According to its anti-bacterial efficacy, we, therefore, hypothesized that hydroquinine might show disinfection efficacy on contact lenses when included as part of commercial MPSs. To validate the hypothesis, we compared the disinfection efficiency of all tested solutions via anti-bacterial activity, anti-adhesion efficacy, and anti-biofilm mass/formation on a contact lens.

In the present study, we observed the efficacy of Opti-free^®^ Replenish^®^ solution (MPS A) and Q-eye multipurpose solution (MPS B), which are available for sale in Thailand. It was discovered that they had the anti-bacterial capacity against the growth of both representatives from standard and clinical *P. aeruginosa* strains according to ISO 14729 criteria (Figure 2). Comparing MPSs, hydroquinine and its combination also reduced the growth of *P. aeruginosa* strains with similarity in reduction rates at more than 3 log of reduction (Figure 3). This is the first report that hydroquinine was as effective as commercially available MPSs. Therefore, hydroquinine might be used as a disinfecting contact lens solution like MPSs for inhibiting bacterial growth. Moreover, both MPSs also reduced the bacterial adhesion on contact lens surfaces. It is interesting to note that MPS A displayed greater disinfection efficacy than MPS B. Comparing the disinfectant agents, MPS A is composed of two biocides including 0.001% polyquaternium-1 (PQ-1, as the predominant anti-bacterial agent [50]), and 0.0005% myristamidopropyl dimethylamine (MAPD, as a broad spectrum antimicrobial agent [50]). However, MPS B contains only 0.0001% polyhexamethylene biguanide (PHMB, as the anti-bacterial agent [51]). PQ-1 and PHMB are agents in the family of quaternary ammonium compounds (QACs) [51]. The QACs interact with the bacterial outer membrane and then induce cytoplasmic membrane damage, resulting in the loss of membrane integrity, intracellular component leakage, and cell lysis [50,52]. However, MPS A contains more ingredients, e.g., MAPD, and the dual biocides may be a reason why MPS A has more antimicrobial activity than MPS B, which is consistent with the work of De Azevedo Magalhaes et al. [53]. The higher concentrations of the QACs in MPS A may also explain the increased disinfection efficacy.

Interestingly, we found that at half of their original concentration, the MPSs were still able to inhibit growth and reduce adhesion. Previous research reported that the manufacturer’s solutions at their original concentration (100%) containing biocides may cause some ocular adverse effects [54]. To minimize the risk of ocular complications, we hypothesized that half the original concentration of MPS A should have enough disinfection efficacy when combined with or without the hydroquinine on contact lenses. We demonstrated that hydroquinine with commercial MPS showed synergistic effects, reducing *P. aeruginosa* adhesion on contact lens surfaces and limiting biofilm formation. Our research suggests that hydroquinine suppresses *P. aeruginosa* fimbrial activity by impairing surface attachment and interrupting their chemotaxis, resulting in the prevention of biofilm formation. This is supported by previous research showing that hydroquinine could suppress L-arginine via the arginine deiminase pathway, resulting in decreased biofilm formation [31]. This is also consistent with a previous study by Rattanachak et al. showing that hydroquinine could suppress QS-related gene expression, reduce virulence factor production, and impair biofilm formation in *P. aeruginosa* [32].

A previous in vitro study demonstrated that commercially available disinfecting solutions were not effective against biofilms [20,21]. In this study, it is demonstrated that the *P. aeruginosa* biofilm mass can be efficiently eradicated by either hydroquinone alone or in combination with commercially available MPSs. The results indicate that the combinations were effective in inhibiting the formation of biofilm on the external surface of the contact lens.

The present study is the first strong evidence that the effectiveness of MPS combined with hydroquinine can inhibit *P. aeruginosa* adhesion and prevent biofilm formation on contact lens surfaces. Therefore, we suggest that soaking contact lenses in MPS containing hydroquinine is possibly helpful in decreasing bacterial adhesion, preventing biofilm formation, and removing the existing biofilm mass. Further testing may be necessary to assess the safety of MPS formulations containing hydroquinine, thereby minimizing the risk of adverse ocular effects. Nevertheless, hydroquinine exhibits potential for use as part of a disinfectant to prevent bacterial growth on contact lenses. This potential development could contribute to the creation of new disinfectants from natural products, effectively combating *P. aeruginosa* infections and reducing the CLMK incidence. There are some limitations that need to be addressed. In this study, there is limited available evidence regarding the disinfection efficacy of various contact lens materials. This study employed only polymacons. We suggest that, for further investigation, the disinfection efficacy of hydroquinine with several different contact lens types is required. Further work will seek to determine the disinfection efficacy of hydroquinine against other pathogenic microorganisms. Additional environmental conditions may be included in future work, for example, an in vitro model under consumer-use conditions to closely mimic the real situation. Furthermore, the safety assessment of hydroquinine is now challenging. An in vitro cytotoxicity of hydroquinine in human cells and an in vivo in animal models should be investigated in the future to provide useful data before moving forward to clinical trials.

## 4. Materials and Methods

### 4.1. P. aeruginosa Strains, Cultivation, and Inoculum Preparation

*P. aeruginosa* ATCC 27853 was obtained from the American Type Culture Collection (ATCC; Manassas, VA, USA) and a clinical *P. aeruginosa* strain was isolated from an eye-infected and hospitalized patient from a previous study [31]. The bacterial isolates were steaked on the Mueller Hinton Agar (MHA, Oxoid, Basingstoke, UK) and then incubated overnight at 35 ± 2 °C. The turbidity of inoculum was adjusted to 0.5 McFarland standard around 1–2 × 10^8^ CFU/mL [29,31,32].

### 4.2. Contact Lenses and Lens Cases

Sterilized soft contact lenses (Maxim Sofeye; Vision Science Co., Ltd., Gyeongsangbuk-do, Republic of Korea) were purchased. The lenses were U.S. Food and Drug Administration (FDA) group 1, with a 14.1 mm diameter and 8.6 mm base curvature. The hydrogel contact lenses were made from polymacon, which had 2-hydroxyethyl methacrylate (HEMA) as the main monomer (58% HEMA and 42% Water). Contact lens cases were obtained from the manufacturer’s supplies. All contact lenses and lens cases were new and unused before testing.

### 4.3. Commercial Multipurpose Solutions

The two commercial soft contact lens MPSs, which were available in Thailand, were tested. The tested solutions were Opti-free^®^ Replenish^®^ solution (Alcon Laboratories, Inc., Fort Worth, TX, USA), which coded as MPS A, and Q-eye multipurpose solution (Stericon Pharma Pvt. Ltd., Karnataka, India), which coded as MPS B. In this study, the 100% original product was tested, and 50% of the MPS was prepared by dissolving the original product with phosphate buffer saline pH 7.4 (PBS; Sigma-Aldrich, Merck, Darmstadt, Germany). The component of each solution is shown in Table 3.

### 4.4. Solution Preparation

The initial solution of hydroquinine (CAS No. 522-66-7) (Sigma-Aldrich, Merck, Darmstadt, Germany) was prepared in 25% dimethyl sulfoxide (DMSO) in PBS to achieve 20 mg/mL. The working solution of hydroquinine was diluted in PBS to achieve the required concentration. The MIC of hydroquinine (2.50 mg/mL) and half MIC (1.25 mg/mL) employed in this study were from previous studies [31,32]. For all the disinfection efficacy testing, the tested solutions were compared with the control (PBS).

### 4.5. Studying Gene Expression Levels

To verify the adhesion-related gene expression levels, the drug-sensitive *P. aeruginosa* ATCC 27853 was treated with and without hydroquinine. The gene expression steps were as follows: RNA extraction, complementary DNA synthesis, and RT-qPCR, respectively [29,31,32].

#### 4.5.1. RNA Extraction

The RNA extraction procedure was carried out as described in previous publications [29,31,32]. In brief, *P. aeruginosa* was cultured to reach a turbidity of 0.5 McFarland standard. In the treated group, a solution of hydroquinine at a concentration equal to half the MIC (1.25 mg/mL) was administered. Conversely, the untreated group was cultured in Mueller Hinton Broth (MHB, Oxoid, Basingstoke, UK) supplemented with DMSO. Every tube was placed in an incubator at a temperature of 35 ± 2 °C for an hour. Subsequently, the pellet was harvested using the 4 °C centrifugation at 5000 rpm for 10 min. The total RNA presented in the pellet was then isolated using the RNeasy Mini Kit (QIAGEN, Hilden, Germany). Residual DNA was digested by DNase solution. The Microvolume Spectrometer (Colibri LB 915, Titertek Berthold, Pforzheim, Germany) was employed to assess both the purity and quantity of the total RNA samples.

#### 4.5.2. Complementary DNA (cDNA) Synthesis

The cDNA was synthesized using a FIREScript RT cDNA synthesis kit (Solis Biodyne, Tartu, Estonia) as previously documented [29,31,32]. Briefly, the 20 μL reaction was prepared by adding 500 ng of the RNA sample, 100 μM oligo (dT) primers (1 μL), 10 × RT buffer (2 μL), reverse transcriptase (RT; 1 μL), dNTP Mix (0.5 μL), 40 U/μL RNase inhibitor (0.5 μL), and then RNase-free water. The conditions for converting cDNA were as follows: an initial annealing step at 25 °C for 5 min, followed by a reverse transcription step at 45 °C for 15 min, and an RT inactivation step at 85 °C for 5 min. The concentration of cDNA was quantified before further analysis.

#### 4.5.3. Quantitative Reverse Transcription PCR (RT-qPCR)

The RT-qPCR was performed in PCR tubes (Bio-Rad Laboratories, Hercules, CA, USA) using HOT FIREPol^®^ EvaGreen^®^ qPCR Mix Plus (Solis Biodyne, Tartu, Estonia) according to the manufacturer’s protocol. Briefly, specific primers for adhesion-related genes are presented in Table 4. The PCR tubes containing the extracted RNA and qPCR reagent were then positioned in the LineGene 9600 Plus Real-Time PCR Detection System (Bioer Technology, Hangzhou, China), following the RT-qPCR cycle conditions: 40 cycles of denaturation at 95 °C for 15 s, suitable annealing temperature at 56–58 °C for 20 s, and extension at 72 °C for 20 s. The relative fold changes of gene expression levels were calculated against the housekeeping *16S rRNA* gene as mentioned in [29,31,32].

### 4.6. Stand-Alone Testing with Microorganisms

The antimicrobial efficacy of the tested solutions was determined using stand-alone testing with some modifications according to the International Organization for Standardization (ISO) 14729 [19]. ISO 14729 is Ophthalmic optics—Contact lens care products—Microbiological requirements and test methods for products and regimens for hygienic management of contact lenses [19].

Briefly, the antimicrobial effectiveness was performed by inoculating 1.0 × 10^5^ to 1.0 × 10^6^ CFU/mL of each tested *P. aeruginosa* strain into the test tube, which contained 10 mL of each tested solution. The test samples were stored at 20–25 °C. All test samples were assessed to determine the number of surviving bacteria at 6 h (recommended disinfection time) and 24 h (additional time point). To count the number of living bacteria, aliquots of the tested solution (1 mL) were transferred to new test tubes containing 9 mL of MHB. Serial 1:10 dilutions were then performed using additional test tubes containing MHB. Dilutions were then plated to quantify the colony-forming unit (CFU/mL). Plate counts were conducted and calculated to the log of reduction compared to the test control (PBS).

### 4.7. Anti-Adhesion Efficacy of Tested Solutions

The anti-adhesion efficacy was determined using a crystal violet retention assay [32]. Briefly, in 96-well plates, each well containing either 200 µL of tested solution or PBS was challenged with 20 µL of inoculum (approx. 10^5^ to 10^6^ CFU/mL of each *P. aeruginosa* strain). The plates were kept in an incubator at 35 ± 2 °C for 24 h. Subsequently, the planktonic cells were meticulously eliminated, followed by three washes with sterile distilled water (DW). The plates were then subjected to drying at 60 °C for 45 min. Afterward, the adherent cells were stained with 0.1% (*w*/*v*) crystal violet for 20 min at room temperature. The crystal violet stain was thrice rinsed with sterile DW and then re-dissolved in absolute ethanol. The optical density at 595 nm was determined for quantifying the residual biofilm using a microplate reader (PerkinElmer, Waltham, MA, USA) and subsequently calculated as the percentage of anti-adhesion efficacy.

### 4.8. Anti-Adhesion Efficacy on Contact Lens

The antimicrobial effectiveness of the tested solutions on contact lenses was established using ISO 18259 [55] with minor modification. ISO 18259 is a protocol methodology for Ophthalmic optics—Contact lens care products—Method to assess contact lens care products with contact lenses in a lens case, challenged with bacterial and fungal organisms [20,55]. Briefly, contact lenses were aseptically removed from the package and immersed in PBS for 18 h before testing. The lenses were placed with the concave side up in the matching manufacturer’s contact lens cases. Lenses were then inoculated to contain a final count of 1.0 × 10^5^ to 1.0 × 10^6^ CFU/mL of the *P. aeruginosa* tested strains. Following a contact time of 5 min, the required tested solution was added to the cases (4 mL), and the cases were then closed, ensuring the cap was not contaminated. Closed contact lens cases were stored at 20–25 °C for 6 h. PBS was used as the test control and performed in the same manner. Following this time point, the test solutions and control were evaluated to determine the morphology of bacteria at the recommended disinfection time (6 h). The contact lenses were carefully removed from their cases. Next, field emission scanning electron microscopy (FE-SEM; Apreo S, Thermo Fisher Scientific, MA, USA) was used to determine the characterization of *P. aeruginosa* morphology.

### 4.9. Destruction of Biofilm on Contact Lens

To compare the architecture of the biofilm mass in *P. aeruginosa* strains on different tested solutions after the recommended disinfection time (6 h), the anti-biofilm efficacy was performed in the same manner as the previous method with minor modification. Briefly, sterile contact lenses were rinsed with PBS and then placed in 12-well plates containing 1.0 × 10^5^ to 1.0 × 10^6^ CFU/mL of the *P. aeruginosa* tested strain at 35 ± 2 °C for 24 h (biofilm formation phase). Following this, contact lenses were then transferred to new 12-well plates containing the required tested solution (4 mL) and then stored at 20–25 °C for 6 h. As a control, PBS was employed. After this immersion, the morphology of bacterial biofilm mass was examined using the FE-SEM.

### 4.10. Morphological Observations Using the FE-SEM

The morphology of the tested *P. aeruginosa* strains on the contact lens surface was measured using the FE-SEM. For sample preparation, the contact lens samples were cut into 8 mm diameter and put on an aluminum stub. The contact lens was then dehydrated in a desiccator to eliminate the moisture before being coated with gold. At this stage, the contact lens was ready for testing. The FE-SEM measurements were performed at 2.0–10 kV in magnification 1200×, 5000×, and/or 10,000×. The FE-SEM images were used to measure the bacterial morphology including their structure, size, and shape. Based on these characteristics, the morphology was utilized to differentiate between the PBS (control) and tested solutions.

### 4.11. Statistical Analysis

All the tests were carried out in triplicate with three independent repeats. Data were presented as mean ± standard deviation. GraphPad Prism version 8.0.1 (San Diego, CA, USA) was used to analyze the data and generate the graph. One-way analysis of variance (ANOVA) and a Tukey test were used to verify the mean differences between groups. Statistical significance was defined as *p* values less than 0.05.

## 5. Conclusions

This study explored the disinfection efficacy of hydroquinine. Using RT-qPCR, it was determined that hydroquinine influences the expression levels of several genes, namely, *cgrC*, *cheY*, *cheZ*, *fimU*, and *pilV*. Hydroquinine was as effective as commercially available MPSs in terms of anti-*P. aeruginosa* growth and anti-adhesion properties. The effectiveness of MPS combined with hydroquinine in inhibiting *P. aeruginosa* adhesion and destroying *P. aeruginosa* biofilms was demonstrated using the crystal violet retention assay and FE-SEM. This study revealed the novel insights that hydroquinine, as a component in contact lens disinfecting solutions, has the potential for both adhesion inhibition and biofilm destruction. These innovative findings could contribute significantly to the development of new disinfectants that are effective in combating microorganisms, particularly *P. aeruginosa.*

## Figures and Tables

**Table 1:** Anti-adhesion efficacy of multipurpose solutions against *P. aeruginosa* strains.

Tested Solutions	*P. aeruginosa* ATCC 27853	Clinical *P. aeruginosa* Strain
PBS	00.00 ± 0.54	00.00 ± 0.63
50% MPS A	89.87 ± 0.03 *^,a^	89.76 ± 0.07 *^,a^
100% MPS A	91.89 ± 0.19 *^,b^	91.37 ± 0.08 *^,d^
50% MPS B	75.30 ± 1.36 *^,a,c^	75.05 ± 1.58 *^,a,c^
100% MPS B	83.54 ± 1.10 *^,b,c^	86.83 ± 0.90 *^,c,d^

Note: Statistical differences among tested solution families: * is *p* < 0.0001 for each MPS vs. PBS within the same strain, ^a^ is *p* < 0.0001 for 50% MPS A vs. 50% MPS B within the same strain, ^b^ is *p* < 0.0001 for 100% MPS A vs. 100% MPS B within the same strain, ^c^ is *p* < 0.0001 for 50% MPS B vs. 100% MPS B within the same strain, and ^d^ is *p* < 0.01 for 100% MPS A vs. 100% MPS B within the same strain.

**Table 2:** Anti-adhesion efficacy of hydroquinine (HQ) solutions in combination with MPSs against *P. aeruginosa* strains.

Tested Solutions	*P. aeruginosa* ATCC 27853	Clinical *P. aeruginosa* Strain
PBS	00.00 ± 0.54	00.00 ± 0.63
HQ [1.25] in PBS	57.80 ± 0.41 *^,A^	54.84 ± 3.76 *^,C^
HQ [1.25] with 50% MPS A	95.59 ± 0.13 *^,a^	92.39 ± 0.55 *^,c^
HQ [1.25] with 100% MPS A	97.91 ± 0.21 *^,a^	93.64 ± 0.17 *^,c^
HQ [2.50] in PBS	59.56 ± 0.34 *^,B^	56.37 ± 1.27 *^,D^
HQ [2.50] with 50% MPS A	96.49 ± 0.22 *^,b^	92.23 ± 0.16 *^,d^
HQ [2.50] with 100% MPS A	97.16 ± 0.03 *^,b^	93.31 ± 0.20 *^,d^

Note: Statistical differences among tested solution families: * is *p* < 0.0001 for each tested solution vs. PBS within the same strain, ^Aa^, ^Bb^, ^Cc^, and ^Dd^ are *p* < 0.0001 for comparing the hydroquinine alone (uppercases) with the combination (lowercases) at the same hydroquinine concentration and within the same strain.

**Table 3:** Commercial multipurpose solutions (MPSs) were used in this study.

Code	MPS A	MPS B
Product	Opti-free^®^ Replenish^®^ solution	Q-eye multipurpose solution
Manufacturer	Alcon Laboratories, Inc., Fort Worth, TX, USA	Stericon Pharma Pvt. Ltd., Karnataka, India
Disinfectants and preservatives	polyquaternium-1—POLYQUAD^®^ 0.001%, myristamidopropyl dimethylamine—ALDOX^®^ 0.0005%	polyhexamethylene biguanide (PHMB) 0.0001%
Wettings agents	poloxamine—TETRONIC^®^ 1304	poloxamer, dexpanthenol
Lubricants	propylene glycol	sorbitol
Buffer and saline	sodium citrate, sodium borate, sodium chloride	disodium edetate, trometamol, sodium dihydrogen phosphate dihydrate

**Table 4:** Specific primer sequences and annealing temperatures were used in this study.

Genes	Primer Direction	Oligonucleotide Sequences (5′ to 3′)	Annealing Temperature (°C)	References
*cgrC*	Forward	CGAGCGGATTGAAGCCATC	57	This study
	Reverse	ACGATGGGCTGGGTGAATC		This study
*cheZ*	Forward	ACTGGTGGACTGTCTCGAAC	57	This study
	Reverse	CGATCTGCGACATTTCCTGC		This study
*cheY*	Forward	CCACGATGAGACGCATCATC	56	This study
	Reverse	ATGTTCCAGTCGGTGACGAG		This study
*fimU*	Forward	GGAACTCAATGCGATGCTGC	56	This study
	Reverse	GAAGGTCAGATGTTCCACGG		This study
*pilV*	Forward	ACGACGTCAAGGACCAGATG	57	This study
	Reverse	GGCAGTTCGTTCTTCACCTG		This study
*16s rRNA*	Forward	CATGGCTCAGATTGAACGCTG	58	[29]
	Reverse	GCTAATCCGACCTAGGCTCATC		[29]

## Data Availability

The data supporting the current study are available from the corresponding author upon request.

## Supplementary Materials

The following supporting information can be downloaded at: https://www.mdpi.com/article/10.3390/antibiotics13010056/s1, Table S1: Differentially expressed genes (DEGs) associated with adhesion as determined by transcriptome analysis.
