# Supplementary material for: Hydroquinine Enhances the Efficacy of Contact Lens Solutions for Inhibiting Pseudomonas aeruginosa Adhesion and Biofilm Formation
Source: Antibiotics (Basel). 2024 Jan 5;13(1):56. doi: 10.3390/antibiotics13010056 (PMC10812619; doi:10.3390/antibiotics13010056)
Supplement: Supplementary file 1 [file antibiotics-13-00056-s001.zip › antibiotics-2807365-supplementary.pdf]

Table S1. Differentially expressed genes (DEGs) associated with adhesion as determined by transcriptome analysis [1].

Gene name	Product name	Log_2 FC[1]	FDR[2]	*p*-value
cgrC	cupA gene regulator C, CgrC	-2.48	1.57×10^{-2}	5.00×10^{-4}
cheY	two-component response regulator CheY	-2.16	4.13×10^{-2}	1.80×10^{-3}
cheZ	chemotaxis protein CheZ	-2.46	2.26×10^{-2}	7.00×10^{-4}
fimU	type 4 fimbrial biogenesis protein FimU	-2.39	2.57×10^{-2}	9.00×10^{-4}
pilV	type 4 fimbrial biogenesis protein PilV	-2.27	3.55×10^{-2}	1.40×10^{-3}

[1]Log_2 FC, Log_2 relative fold changes of the gene expression levels in response to hydroquinine, compared to the untreated control. [2] FDR, false discovery rate showed statistical significances.

Reference

1. Rattanachak, N.; Weawsiangsang, S.; Daowtak, K.; Thongsri, Y.; Ross, S.; Ross, G.; Nilsri, N.; Baldock, R.A.; Pongcharoen, S.; Jongjitvimol, T.; et al. High-throughput transcriptomic profiling reveals the inhibitory effect of hydroquinine on virulence factors in *Pseudomonas aeruginosa*. *Antibiotics.* **2022**, *11*, 1436, doi:10.3390/antibiotics11101436.
